# Early Detection of Lung Cancer: A Review of Innovative Milestones and Techniques

**DOI:** 10.3390/jcm14217812

**Published:** 2025-11-03

**Authors:** Faisal M. Habbab, Eric L. R. Bédard, Anil A. Joy, Zarmina Alam, Aswin G. Abraham, Wilson H. Y. Roa

**Affiliations:** 1 Division of Radiation Oncology, Department of Oncology, University of Alberta, Edmonton, AB T6G 2B7, Canada; 2 Division of Thoracic Surgery, Department of Surgery, University of Alberta, Edmonton, AB T6G 2B7, Canada; 3 Division of Medical Oncology, Department of Oncology, University of Alberta, Edmonton, AB T6G 2B7, Canada

**Keywords:** lung cancer, screening, early detection, innovative techniques

## Abstract

Lung cancer is the most frequently diagnosed cancer and the leading cause of cancer death worldwide. Early detection of lung cancer can lead to identification of the cancer at its initial treatable stages and improves survival. Low-dose CT scan (LDCT) is currently the gold standard for lung cancer screening in high-risk individuals. Despite the observed stage migration and consistently demonstrated disease-specific overall survival benefit, LDCT has inherent limitations, including false-positive results, radiation exposure, and low compliance. Recently, new techniques have been investigated for early detection of lung cancer. Several studies have shown that liquid biopsy biomarkers such as circulating cell-free DNA (cfDNA), microRNA molecules (miRNA), circulating tumor cells (CTCs), tumor-derived exosomes (TDEs), and tumor-educated platelets (TEPs), as well as volatile organic compounds (VOCs), have the power to distinguish lung cancer patients from healthy subjects, offering potential for minimally invasive and non-invasive means of early cancer detection. Furthermore, recent studies have shown that the integration of artificial intelligence (AI) with clinical, imaging, and laboratory data has provided significant advancements and can offer potential solutions to some challenges related to early detection of lung cancer. Adopting AI-based multimodality strategies, such as multi-omics liquid biopsy and/or VOCs’ detection, with LDCT augmented by advanced AI, could revolutionize early lung cancer screening by improving accuracy, efficiency, and personalization, especially when combined with patient clinical data. However, challenges remain in validating, standardizing, and integrating these approaches into clinical practice. In this review, we described these innovative milestones and methods, as well as their advantages and limitations in screening and early diagnosis of lung cancer.

## 1. Introduction

Lung cancer is the most frequently diagnosed malignancy and the leading cause of cancer deaths worldwide, with an estimated 2.5 million new cases and 1.8 million deaths in 2022 [1]. Most lung cancer statistics include both small-cell lung cancer (SCLC) and non-small-cell lung cancer (NSCLC). SCLC accounts for about 13% of lung cancers and is known for its aggressive growth. NSCLC is the most common subtype, accounting for about 87% of all lung cancer cases, which makes it highly prevalent with high mortality rates [2]. Early-stage lung cancer rarely has specific or clear symptoms, which can delay diagnosis and effective treatment. Early detection of lung cancer can lead to identification of the cancer at its initial treatable stages and improves the 5-year survival, knowing that the 5-year survival in stage IA is around 82% and drops significantly to about 7% in stage IVB patients [3]. Therefore, efforts have been made to improve early diagnosis and treatment of lung cancer to improve patient outcomes. Chest X-ray (CXR) and sputum cytology were used in the 1970s to detect early lung cancer, but they were not found to be effective in reducing its mortality [4,5]. Then, low-dose chest computed tomography (LDCT) was described in the 1990s and was shown to be useful in lung cancer and to be superior to CXR for observational studies [6]. The National Lung Screening Trial (NLST) and Nederlands-Leuvens Longkanker Screenings Onderzoek (NELSON) trial have provided evidence that lung cancer screening (LCS) of high-risk individuals using LDCT reduces the mortality of lung cancer by over 20% [7,8]. This was achieved by shifting the diagnosed stage to an early, potentially curable stage [9]. Currently, guidelines recommend LDCT for individuals ≥50 years old with ≥20 pack-year history or ≥20-year history of smoking cigarettes [10]. Despite its proven mortality-lowering effect, LDCT has inherent limitations, including false-positive results that can lead to unnecessary testing and invasive procedures with increased cost and reduced quality of life because of associated significant anxiety and distress; false-negative results that may delay or prevent diagnosis and treatment due to a false sense of good health; overdiagnosis of indolent disease that will lead to unnecessary therapy; cumulative radiation exposure from repeated annual scans and additional follow-up scans; incidental lesions; and financial costs that can be challenging in modern economic times [11,12,13,14].

Several new techniques utilizing liquid biopsy biomarkers [15,16] and volatile organic compounds (VOCs) [17] have emerged as powerful tools of investigation that can differentiate lung cancer patients from healthy subjects, offering potential for early detection of lung cancer. Also, the integration of artificial intelligence (AI) with clinical, imaging, and laboratory data has significantly enhanced the diagnostic accuracy of these techniques and provided significant advancements in lung cancer screening by improving efficiency and personalization [18]. The aim of this review is to describe these milestones and techniques, as well as their advantages and limitations in screening and early diagnosis of lung cancer.

## 2. Low-Dose Computed Tomography (LDCT)

Screening for the early detection of lung cancer by CXR was not found to be effective in reducing mortality [4,5]. Therefore, substantial attention was directed towards CTs. Conventional chest CT could not be used in screening for lung cancer because its effective radiation dose is ~7.0 millisievert (mSv), which is 70 times more than that of CXR and studies have shown that current CT imaging might contribute to future cancer cases [19]. On the other hand, LDCT has become a well-recognized method for the early detection of lung cancer, given its low effective radiation dose of ~1.5 mSv [20]. It became the most common method of early screening for lung cancer and was found to be superior to CXR in observational studies [6]. Nevertheless, lung cancer screening utilizing LDCT for the general population poses a prohibitive burden on health care systems. Therefore, lung cancer screening by LDCT for high-risk populations was developed; the identification of which is very important in order to derive the greatest advantages.

### 2.1. Identification of High-Risk Clinical/Epidemiologic Status

Lung cancer screening by LDCT starts with risk assessment of asymptomatic individuals for lung cancer. Although age and history of smoking have been shown to have a great influence on the likelihood of developing lung cancer [21], other potential risk factors for lung cancer, including occupational exposure (to substances such as asbestos, arsenic and cadmium), radon exposure, cancer history (lymphomas, cancers of the head and neck, or smoking-related cancers), family history of lung cancer (in first-degree relatives), and lung disease history (COPD or lung fibrosis), should be considered while identifying high-risk candidates for lung screening [22]. This can be facilitated by using risk calculators, such as the Tammemägi lung cancer risk calculator [23,24].

Most of the risk prediction models used for lung cancer are based on age and smoking status (pack-years as a summary measure of smoking exposure) [25,26]. The NLST screening study included current and former heavy smokers (≥30 pack-years smoking history and <10 years ex-smoker) age 55–75 and randomized 53,454 participants to undertake three annual screenings with chest LDCT or posteroanterior CXR [7]. The study reported a 20% decrease in lung cancer-related mortality (HR = 0.80; *p* < 0.004) after 6.5-year follow-up and a noticeable relative decrease of 6.7% (95% CI 1.2–13.6, *p* = 0.02) in all-cause mortality in the LDCT group [7].

**Figure 1:**
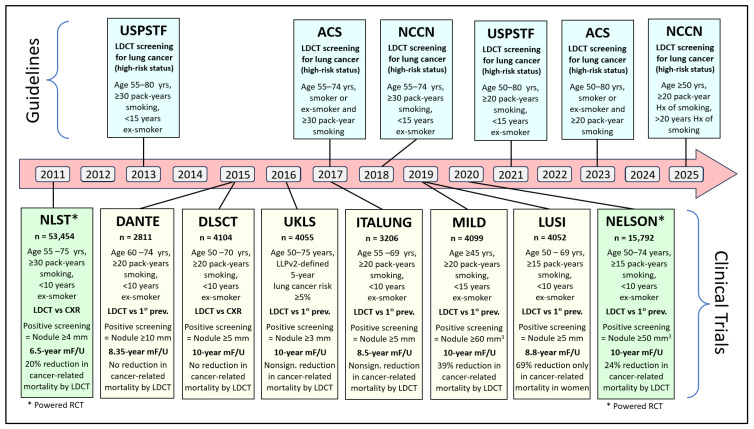
Timeline of main guidelines and major milestone randomized controlled trials (RCTs) of low-dose computed tomography (LDCT)-based lung cancer screening. (ACS = American Cancer Society, DANTE = Detection And screening of early lung cancer with Novel imaging Technology, DLCST = Danish Lung Cancer Screening Trial, ITALUNG = Italian Lung study, LUSI = German Lung Cancer Screening Intervention, MF/U = mean follow-up, MILD = Multicentric Italian Lung Detection trial, NCCN = National Comprehensive Cancer Network, NELSON = Nederlands-Leuvens Longkanker Screenings Onderzoek, NLST = the National Lung Screening Trial, UKLS = UK Lung Cancer Screening, USPSTF = United States Preventive Services Task Force).

The United States Preventive Services Task Force (USPSTF) made a recommendation in 2013 for annual screening of lung cancer with LDCT in asymptomatic adults aged 55 to 80 years who have a 30 pack-year smoking history and currently smoke or have quit smoking within the past 15 years [27]. Similar recommendations were made after that by the American Cancer Society (ACS) and the National Comprehensive Cancer Network (NCCN) (Figure 1) [28,29]. Following NLST, six non-powered randomized clinical trials studied cancer-related mortality by LDCT screening (Figure 1) [30,31,32,33,34,35]. Four showed no or nonsignificant reduction in cancer-related mortality by LDCT screening [30,31,32,33,34,35], one showed a significant 39% reduction in all patients [34], and one showed significant reduction only in women [35] (Figure 1). Five trials identified high-risk populations using age and smoking status (Figure 1), while the UK Lung Cancer Screening Trial (UKLS) [32] used the Liverpool Lung Project version 2 (LLPv2) risk model criteria that define a 5-year lung cancer risk ≥5% in 50–75-year-old individuals [36] (Figure 1). In 2020, the NELSON trial randomized 15,792 subjects (age 55–75 years, ≥15 pack-years, <10 years ex-smoker) to have LDCT screening (at baseline, 1, 3, and 5 years) or no intervention. The study showed a 24% lung cancer-related mortality in the screening arm (HR = 0.76; *p* < 0.004) after 10-year follow-up [8] (Figure 1). A meta-analysis of all seven trials that used age and smoking status to identify high-risk populations to be screened by LDCT enrolled 84,558 participant patients with a smoking history of >15 pack-years and reported a significant 17% relative reduction in lung cancer mortality and a 4% reduction in all-cause mortality in the LDCT screening group compared with the control group [37]. Accordingly, LDCT has been recognized as the standard screening method for lung cancer in high-risk populations as defined by the updated guidelines of USPSTF, ACS, and NCCN (Figure 1) [22,38,39].

### 2.2. Defining Positive Screening Examination on LDCT

A positive screening examination is usually defined by lung nodule size assessed by measuring its greatest transverse diameter or its volume. In the NLST study, a measurement of 4 mm in greatest transverse diameter was used as a threshold to define the positive LDCT screening examination [7]. Using this value, 96.4% of the 24.2% of people who had positive tests were false positives and did not result in a diagnosis of lung cancer in the study [7]. Such a high false-positive rate can lead to unnecessary patient anxiety, radiation exposure, and the risk of an additional invasive workup to rule out lung cancer definitively [40]. Further examination of the NLST data showed that raising the nodule size threshold for a positive screen would markedly reduce false-positive LDCT screenings and the potential unnecessary risks associated with them [41]. Also, further evaluation of data from 134 NLST participants showed that computer-aided diagnosis can improve the reader agreement on the positivity of screening results and follow-up [42]. Similar to the NLST study, the greatest transverse diameter was used for lung nodule size assessment (Figure 1). However, with the availability of thin-slice CT with three-dimensional segmentation software, the semi-automated volumetric approach has become more effective, being more accurate and more reproducible [43,44].

The NELSON trial used volume LDCT lung cancer screening and introduced the concept of “indeterminate” screening classification, which resulted in a significant reduction in false-positive screens (only 1.2% out of 43.5% positive screens) and a significant reduction in unnecessary workup procedure risks without affecting favorable outcomes [8]. Also, volumetry is very useful in the assessment of growth, which is key to assessing malignancy. Volume doubling time (VDT) is more accurate than the increase in diameter in the assessment of cancer growth, as a 100% increase in volume, i.e., VDT, results only in a 26% increase in diameter [45]. Therefore, volume measurements have been included in the Lung CT Screening Reporting and Data System (Lung-RADS) and are preferred over diameter measurements for nodule size assessment and growth determination in LDCT lung cancer screening [46].

### 2.3. Nodule Management

In the NELSON study, nodule management incorporated a nodule assessment approach that includes nodule size and its volumetric assessment, growth rate, subtype (solid, part-solid, or non-solid), and their associated malignancy risk [8]. The majority of lung cancer screening programs, including the NCCN, ACS, USPSTF, and European Society of Thoracic Imaging (ESTI), now use Lung-RADS as the way to triage the next investigation or step to take for patients with nodules [22,38,39,47]. Lung-RADS was created by the American College of Radiology (ACR) as a quality assurance tool to standardize the way of reporting and managing lung nodules detected during LDCT lung cancer screening to improve patient outcomes and to decrease confusion in screening programs. It classifies findings into categories based on nodule characteristics (solid, sub-solid, mixed density, and atypical lung cysts) and provides directions for next steps (follow-up imaging or diagnostic evaluations). Categories 1 or 2 indicate negative results that require no further action. Category 3 suggests a low likelihood of malignancy that requires short-interval follow-up imaging. Category 4 (including 4A, 4B, and 4X) indicates a higher suspicion of malignancy and requires more urgent diagnostic evaluation or follow-up [48].

A solid nodule in the NELSON study was defined during baseline screening as negative when its volume was <50 mm^3^, indeterminate when its volume was 50–500 mm^3^, and positive when the volume was >500 mm^3^ [8]. In addition, growth rate and associated malignancy risk play a major role in the management of lung nodules during repeat rounds of screening [8]. Table 1 summarizes current (2025) screening recommendations of the NCCN and the European Society of Thoracic Imaging (ESTI) for management of solid nodules [22,47]. Both guidelines focus on the management of nodules found on LDCT scans. The NCCN categorizes solid nodules based on risk, nodule size (diameter), and characteristics, which dictate the recommended follow-up schedule, typically at 6–12 months, to balance the risk of cancer with minimizing unnecessary procedures. On the other hand, ESTI recommendations focus on lesion aggressiveness, using nodule volume doubling time (VDT) and morphology to determine follow-up at 3–6 months, which aims to reduce the number of unnecessary follow-up CT scans while preventing advanced-stage detection and overtreatment.

### 2.4. Advantages and Limitations

The aim of early detection of lung cancer is to identify the cancer in its treatable and curable stages. The potential tremendous advantages of lung cancer screening by LDCT include the following: (i) Oncology outcome advantages. Powered randomized controlled trials showed a significant reduction in lung cancer mortality of over 20% [7,8]. (ii) Quality of life advantages. Both the NLST and the NELSON trials assessed quality of life among participants at the time of the LDCT screening study [49,50], demonstrating a reduction in disease-related and treatment-related morbidity and in anxiety and psychological burden. It is possible that quality-of-life advantages are explained by early lung cancer detection (as opposed to detection at the time of clinical symptoms) and by negative LDCT findings. (iii) Discovery of other significant occult health risks (e.g., thyroid nodule, breast cancer, etc.) [22].

The limitations associated with lung cancer screening by LDCT include the following: (i) False-positive results, which can lead to unnecessary testing and invasive procedures; increased cost because of these added follow-up tests; and reduced quality of life because of mental suffering associated with false-positive screening results that can cause significant anxiety and distress for patients while they await further diagnostic tests. (ii) False-negative results, which may delay or prevent diagnosis and treatment because of a false sense of good health. LDCT scans can miss some fast-growing lung cancers or those located in difficult-to-image areas. Also, aggressive “interval“ cancers can develop rapidly between annual screening scans and may have worse outcomes. In addition, studies have shown that a high percentage of patients whose cancer was missed on an LDCT scan were eventually diagnosed with advanced-stage disease and had a high mortality rate. (iii) Overdiagnosis of indolent disease, which is not harmful to the patient but will lead to unnecessary therapy. (iv) Indeterminate results, leading to additional testing. (v) Cumulative radiation exposure from repeated annual scans and additional follow-up scans for suspicious findings that may require a higher dose of radiation. (vi) Physical complications from diagnostic workup. (vii) Incidental lesions. (viii) Anxiety about test findings that will affect quality of life. (ix) The financial costs can be challenging in modern economic times when healthcare resources are scarce and often torn among competitive demands [13,14,51,52]. There is a possibility of missing lung cancer in non-smokers, especially in Asian countries and particularly in younger patients and women, with increased incidence of lung adenocarcinoma. This is possibly due to outdoor air pollution (from vehicles, industrial activities, and power plants), indoor air pollution (especially cooking oil fumes), genetics, and female hormones [53,54]. Recent reports suggest that the integration of liquid biopsy biomarkers in the screening programs could help identify these patients [55].

### 2.5. Future Perspectives

Because of the limitations of LDCT, improvements in the current lung cancer screening pattern are needed. Combination with other techniques, such as EB-OCT combined with CT screening and with needle biopsy, circulating biomarkers, and integration of artificial intelligence (AI), can offer potential solutions to some of these challenges. These techniques could be ideal complements to LDCT, improving sensitivity and specificity and decreasing the rate of false positives. In addition, recent advances in CT hardware technology and sophisticated image reconstruction algorithms have led to the introduction of ultra-low-dose CT (ULDCT), which has a very low radiation dose with the potential of significantly reducing radiation exposure [56,57,58]. Recent studies have shown that common ULDCT doses range from 0.13 to 0.49 mSv and that it has the potential of safely and effectively detecting and evaluating lung nodules [57,58,59]. Awaiting further clinical validation and refinement of low-dose techniques, ULDCT is expected to have great potential for lung cancer screening and management in daily clinical practice [60].

## 3. Liquid Biopsy Biomarkers

**Figure 2:**
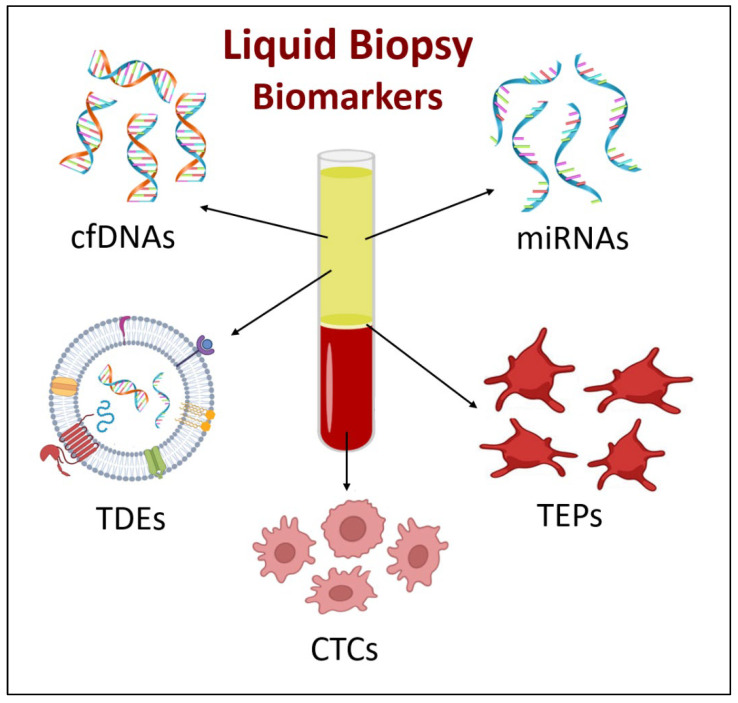
Liquid biopsy biomarkers. Arrows indicate the biomarker’s corresponding level of blood. (cfDNA = circulating cell-free DNAs, CTCs = circulating tumor cells, miRNAs = circulating microRNAs, TDEs = tumor-derived exosomes, and TEPs = tumor-educated platelets).

Liquid biopsies taken from a body fluid can reflect the full state of the tumor and many malignant features utilizing a less invasive, less costly, and easily attainable method [61,62]. When used as an initial screening tool in high-risk smokers, liquid biopsy might identify individuals who require thorough clinical workup, which will reduce LDCT-based false positives [63]. With the advancement of new detection technologies, liquid biopsy is expected to enhance the accuracy and specificity of early detection by improving the currently used strategies, either alone or as complementary tests for lung cancer diagnosis. This technique allows for the detection of several biomarkers that are involved in the detection of lung cancer, including circulating cell-free DNA, circulating tumor cells, circulating microRNAs, tumor-derived exosomes, and tumor-educated platelets [64] (Figure 2).

### 3.1. Cell-Free DNA (cfDNA) and Circulating Tumor DNA (ctDNA)

Circulating cell-free DNA (cfDNA) is a broad term encompassing all DNA fragments of about 100 to 200 base pairs (bp) in length circulating in the bloodstream, including those from healthy normal leukocytes and stromal cells, as well as from diseased cells. The cfDNAs are released into the bloodstream or other related body fluids by apoptosis, necrosis, or active vesicle transport [65]. It was found that plasma levels of cfDNA were significantly increased in patients with advanced tumors compared to healthy subjects, suggesting that it may also be produced by tumor cells [66]. Circulating tumor DNA (ctDNA) is a type of circulating extracellular cfDNA that originates from tumor cells and can be extracted from the bloodstream [67]. It is shorter than cfDNA, being about 20–50 base pairs in length, and accounts only for a small percentage of total cfDNA concentration and has a shorter half-life [68,69]. In addition to being traditionally extracted from blood, ctDNA can also be detected in urine, pleural fluid, cerebrospinal fluid (CSF), and ascites [68]. Being fragments of tumor cell DNA, ctDNA genetic abnormalities are identical to those of the primary tumor, making them ideal for cancer diagnosis. However, they are found in a very small concentration during the early stages of the cancer, because of which their role in early cancer detection has not proven helpful [70]. On the other hand, ctDNA has proven to be very helpful for prognosis, treatment outcomes, and disease progression monitoring in cancer patients [70].

Several studies have addressed the use of cfDNA concentration for early detection of NSCLC. Some failed to show favorable results [71,72], while others found that lung cancer patients have higher cfDNA levels than healthy controls with a proposed cut-off value of 2.8 ng/mL to discriminate between both [73,74]. Advances in the detection technologies of cfDNA enabled its use as part of liquid biopsy for early detection of cancer. The potential use of cfDNA for early detection of lung cancer has been addressed by several studies focusing on its concentration, genetic mutation, or epigenetic alterations (specifically methylation patterns).

Genetic mutations in the epidermal growth factor receptor (*EGFR*) are among the most studied genetic mutations in lung cancer. They have been considered as markers of the efficacy of target therapies and recently as markers of early detection of lung cancer. In one study they showed a 100% concordance rate with nodule biopsy and a global specificity of 95% [75]. In another study, it was demonstrated that DNA isolated from extracellular vesicles was better than cfDNA for mutation detection among patients with early-stage NSCLC [76]. In addition to these single genetic mutations, other studies suggested that whole exome sequencing has the potential for early lung cancer detection in patients with CT-identified pulmonary lesions [77].

DNA methylation is one of the most studied epigenetic mechanisms. It consists of adding a methyl group (CH3) to the 5-carbon position of the cytosine ring within CpG dinucleotides. Normally, DNA methylation plays a vital role in gene expression regulation, genomic stability maintenance, and repetitive element silencing [78]. These patterns are often disturbed in cancer, causing abnormal gene expressions such as hypermethylation or hypomethylation [79]. Epigenetic changes are stable and occur in the early stages of cancer, which makes them appropriate biomarkers for early detection of cancer [78,79,80,81]. These changes are reflected by methylation markers carried by cfDNA, which can be detected in blood and sputum by methylation-specific polymerase chain reaction (MSP), digital droplet PCR (ddPCR), and next-generation sequencing (NGS). Methylation of SHOX2 and RASSF1A genes in plasma was found to be associated with early stages of lung cancer, indicating that blood-based assays have potential for non-invasive screening [82,83]. When combined, gene methylation and clinical information correctly predicted lung cancer in 91% of subjects using sputum and in 85% using plasma [84].

Advantages of using cfDNA and ctDNA as a screening tool for lung cancer include the following: (i) Genetic mutation and epigenetic alterations reflect those of the original tumor [78,79,80,81]. (ii) Epigenetic changes are stable and occur in the early stage of cancer and represent tumor heterogeneity and dynamics [78,79,80,81]. (iii) Availability of highly sensitive assays (PCR, NGS) [82,83]. On the contrary, there are some important potential limitations to the use of cfDNA and ctDNA in early detection of lung cancer: (i) cfDNA is increased in some benign conditions such as cardiovascular diseases, other lung diseases, and infections [85,86,87,88]. (ii) Utilization of this method is associated with high costs [89]. (iii) The need for standardization of laboratory protocols and assays to achieve consistent and reproducible results. (iv) Individuals with early-stage malignancies have lower plasma levels of ctDNA [90] (Table 2).

### 3.2. MicroRNA (miRNA)

Cell-free microRNAs (miRNAs) are single-stranded non-coding fragments that can be detected in the blood of lung cancer patients and play a significant role in gene expression regulation. They are released into the blood and surrounding anatomical tissues through apoptosis, exosomes, and tumor-educated platelets (TEPs) [91]. They are involved in cell development, differentiation, and proliferation and apoptosis by targeting messenger RNAs (mRNAs) and blocking their regulatory effect [92,93]. Oncogenes and tumor suppressor genes can be targets for some of these miRNAs. Abnormally increased miRNA concentrations can lead to tumorigenesis through oncogene activation and/or loss of tumor suppression [94]. The stability of miRNAs in body fluids (serum and plasma) makes them promising biomarkers for early detection of cancer [95].

Between the years 2008 and 2022, studies have shown that different miRNA panels can differentiate lung cancer patients from healthy individuals using miRNAs in serum or plasma (in 20 studies) with a sensitivity of 71% to 95% and a specificity of 61% to 100% [96,97], and miRNAs in sputum or bronchoalveolar lavage (in 11 studies) with a sensitivity of 64% to 86% and a specificity of 80% to 100% [97,98,99,100]. Serum miRNA marker assays allow the detection of asymptomatic disease or may help diagnosis when combined with LDCT [101]. Body fluid markers such as miRNAs-25, 223, 141, 155, and 1254 are involved in the early diagnosis of lung cancer [102]. Other miRNA markers from different body fluids have the potential for early detection of NSCLC, such as serum miRNAs-125a-5p, 25, and 126 [103]; serum and urine metabolites miRNAs- miR-21 and miR-223 [96]; and sputum and bronchoalveolar lavage miRNAs- miR-21, miR-143, miR-155, miR-210, and miR-372) [98,99,100]. The clinical use of a plasma miRNA signature classifier (MSN) based on 24 miRNA expression ratios within CT in lung cancer screening improved the accuracy of LDCT and showed a five-fold reduction in its false-positive rate [104]. Also, MSN achieved a sensitivity of 87% and specificity of 81% for lung cancer detection, compared to the sensitivity and specificity of 88% and 80%, respectively, for the LDCT [104]. In addition, the combination of MSN and LDCT in the BioMILD trial improved the accuracy of lung cancer risk and mortality prediction in heavy smokers and reduced false positives in LDCT screening [105].

Advantages of using miRNA in early detection of lung cancer include the following: (i) Different profiles among early-stage cancer patients. (ii) Stable in most types of body fluids (e.g., respiratory samples). (iii) Commercial kits available for collection [91,106]. Although utilizing miRNAs as a novel and less invasive technique for the early detection of NSCLC is quite encouraging, there are several limitations to their clinical utility: (i) High variability, according to patients and technologies, requiring normalization methods. (ii) Quantification and detection methods need to be validated. (iii) Unspecific for cancer subtype. (iv) RNA’s molecular diagnostic accuracy for nonmalignant cases of the lung was not evaluated (Table 2) [106,107].

### 3.3. Circulating Tumor Cells (CTCs)

Circulating tumor cells (CTCs) are neoplastic cells that detach from the primary tumor and enter the circulatory system [108]. These cells use an epithelial-mesenchymal transformation (EMT) process to penetrate the basal membrane [109]. In the peripheral bloodstream or the lymphatic system, CTCs can be found as single cells or clusters, as well as in clusters with white blood cells, and they can form secondary tumors at new metastatic sites [110,111]. These cells have a similar morphology to primary tumor cells, and their detection is important for the early diagnosis of cancer [112]. The concentration of CTCs in peripheral blood is very low, particularly in the early stages of NSCLC; therefore, the direct separation of these cells from other circulating cells in peripheral blood is challenging [113]. However, with the continuous development of separation technologies, CTCs are expected to become effective biomarkers for early detection of lung cancer, especially because they can be detected earlier than symptoms and before the disease becomes evident by imaging [114].

The use of CTCs as a biomarker for early detection of lung cancer was suggested after all CTC-positive chronic obstructive pulmonary disease (COPD) patients developed lung cancer while being followed annually by LDCT for 1–4 years [115]. This finding also suggests that CTCs can be combined with LDCT for lung cancer screening to decrease its false-positive results. Many studies demonstrated that the use of CTC has the potential to differentiate malignant from benign pulmonary nodules with a sensitivity varying from 26.3% to 92.7% and a specificity varying from 50.0% to 100.0%, depending on the detection and staining method [116]. The combination of CTCs and cfDNA methods significantly increased the sensitivity to diagnose primary lung cancer from 72.7% for the cfDNA method alone and 65.7% for the CTCs method alone to 95.0% for both methods combined [117]. Such a combination strategy could enhance early detection of NSCLC. Detecting CTCs could help identify high-risk populations for screening and discriminate between malignant and benign nodules, ultimately enabling earlier diagnosis of lung cancer. The detection rate of CTCs in SCLC is typically higher than in NSCLC upon diagnosis, at which SCLC is often already at an advanced stage. This could be relevant for early detection; however, their utility as an early screening tool for SCLC is still in its infancy [118,119].

Advantages of utilizing CTCs for early detection of lung cancer include the following: (i) Allows morphological and molecular characterization of lung cancer. (ii) High technical testing sensitivity available [112,113]. However, despite the obvious potential for the early detection of lung cancer, this technique has some limitations: (i) Sample sizes are very small, and data are limited in the majority of studies. (ii) CTCs are rare in blood in early-stage lung cancer. (iii) The observed heterogeneity of CTCs. (iv) Lack of standardized techniques that are validated and can be easily accessed and used (Table 2) [120,121].

### 3.4. Tumor-Derived Exosomes (TDEs)

Exosomes are small (30–150 nm in diameter) extracellular lipid bilayer vesicles that are released by several cells and commonly found in different body fluids, including blood, urine, saliva, and breast milk [122,123]. They contain a collection of biomolecules, including RNA (micro, long non-coding, and circular RNAs), DNA (double- or single-stranded), and proteins [124,125]. Exosomes participate in the process of intercellular communication by activating intracellular signaling, either directly through the activation of target cell receptors by their membrane proteins or by releasing their contents into the target cell [126,127]. Tumor cells release larger amounts of exosomes than normal cells, and these exosomes and their content represent an excellent source of information on cancer cells [127]. Since the exosomes of lung cancer cells are produced in large amounts, carry the genetic information of the cancer cell, and can be detected in different body fluids, they can be considered perfect biomarkers for early detection of lung cancer. The exosomal components that hold considerable potential as biomarkers include miRNA, long non-coding RNA (lncRNA), circular RNAs (circRNA), and proteins [124,125].

The promising benefit of specific exosomal miRNAs as biomarkers for early detection of lung cancer has been demonstrated in several studies. In one study, two exosomal miRNAs (miR-146a-5p and miR-486-5p) and four serum miRNAs (miR-21-5p, miR-141-3p, miR-222-3p, miR-486-5p) showed good potential for the early diagnosis of lung cancer, with the suggestion that exosomal miRNAs are more preferable [128]. The combination of both serum miRNAs and exosomal miRNAs was associated with an additional improvement in the diagnosis of lung cancer at early stages, with a sensitivity of 85.42% and a specificity of 92.50% [128]. In another study comparing patients with early-stage NSCLC with healthy subjects, adenocarcinoma-specific and squamous cell carcinoma-specific exosomal miRNAs (either upregulated or downregulated) were identified with a sensitivity and a specificity of 80.65% and 91.67% and of 83.33% and 90.32%, respectively [129]. Other studies also demonstrated that specific serum or plasma exosomal miRNAs have the potential to differentiate patients with early-stage lung cancer from those in healthy subjects with a sensitivity and a specificity reaching up to 96% and 91%, respectively [130,131,132].

Although they cannot encode proteins, lncRNAs (>200 nucleotides long) play critical roles in important biological processes, such as activation of transcription, silencing of chromosomes, nuclear transport, and modification of chromatin [133,134]. Recent studies have shown that exosomal lncRNAs have the potential to be effective biomarkers for the early detection of lung cancer. As a tumor suppressor, exosomal lncRNA growth arrest-specific transcript 5 (GAS5) was found to be downregulated in patients with early-stage lung cancer compared to healthy individuals, and they were found even lower in patients with advanced lung cancer [135]. Yet, exosomal lncRNA 917 (LINC00917) was found to be upregulated in patients with early-stage NSCLC compared to healthy individuals, and it was found even more upregulated in patients with advanced lung cancer [135]. Other exosomal lncRNAs, such as SOX2-OT, LUCAT1, GAS5, and AL139294.1, were also found to have promising potential for early detection of NSCLC [136].

Recently, exosomal circRNAs, which play a vital role in tumorigenesis, have emerged as potential biomarkers for the early detection of NSCLC. The expression levels of exosomal circRNAs circ_0001492, circ_0001439, and circ_0000896 were noticed to be higher in patients with lung adenocarcinoma compared to healthy individuals, and they significantly decreased after surgery [137]. Also, exosomal circRNAs circRAPGEF5 could detect lung adenocarcinoma with a sensitivity of 64.90% and a specificity of 95.60% [138]. Other studies show that specific serum exosomal circRNAs circ_0048856, circ_0070354, circ_004792, circ_0056285, circ_0007761, and circ_0069313 have the potential to differentiate patients with early-stage NSCLC from healthy subjects with high specificity and sensitivity [139,140,141,142].

Exosomal proteins are produced by parental cancer cells, and their detection can be considered valuable for the early detection of lung cancer [143]. One of these proteins is epidermal growth factor receptor (EGFR), which is found in about 80% of exosomes isolated from lung cancer patients and can be detected in early stages of the disease [144]. Also, exosomal active disintegrin and metalloproteinase domain-containing protein 10 (ADAM10) is significantly increased in patients with lung cancer and can efficiently differentiate these patients from healthy individuals [145]. Several other proteins in exosomes derived from blood (serum/plasma) [146,147,148,149,150,151], urine [152,153], and saliva [154,155] are found to be significantly increased in patients with NSCLC compared to healthy individuals, suggesting their potential as diagnostic biomarkers for early detection of NSCLC.

Advantages of utilizing exosomes for early detection of lung cancer include the following: (i) Carrying specific biological information, as they comprise several types of biomarkers such as RNAs, DNAs, and proteins. (ii) Increased/decreased in lung cancer patients. (iii) Stable and accessible in most types of body fluids. (iv) Commercial kits available for isolation [124,125,156,157]. However, in spite of the obvious potential for the early detection of NSCLC, this technique has some limitations: (i) Efficient extraction and purification of exosomes are challenges. (ii) Low repeatability and complex composition of exosomal RNAs. (iii) Require technical standardization and clinical validation to enhance the practical application. (iv) High costs [158,159,160,161].

### 3.5. Tumor-Educated Platelets (TEPs)

Tumor-educated platelets (TEPs) are platelets that are altered and whose RNA and protein content are influenced either directly by contacting tumor cells and/or indirectly following local communication with the tumor microenvironment [162]. While circulating through the body, TEPs play important roles in supporting tumor survival, growth, and dissemination [163]. Carrying tumor-derived molecules, TEPs can be considered valuable potential biomarkers for early tumor detection and monitoring, including lung cancer [163]. Platelets contain a variable range of RNA types that emerged as promising biomarkers for a variety of tumors [164]. TEPs’ messenger RNA (mRNA) can predict tumorigenesis and monitor tumor progression in lung cancer [165,166]. TEPs’ miRNAs can increase NSCLC cell invasion by targeting tumor suppressors [167]. Some TEPs’ small nuclear RNAs (snRNAs) are downregulated in lung cancer patients and act as potential biomarkers for the disease [168]. During the diagnosis of different NSCLC stages, TEP-based detection showed 81% diagnostic accuracy in the early stage of the disease [169]. Other studies demonstrated that using TEP RNA indicators could successfully differentiate NSCLC patients from healthy controls and malignant from benign pulmonary nodules, suggesting the potential of TEP RNA as an indicator of early detection of NSCLC [170,171,172,173,174].

Advantages of utilizing TEPs for early detection of lung cancer include the following: (i) Carry tumor-modified RNA that reflects the tumor cells’ genetic alteration transcripts. (ii) Stable and highly abundant TEPs. (iii) TEPs are easily isolated in a clinical laboratory. (iv) Compatible with RNA-seq and AI pipelines [175,176,177]. Though there are obvious potential benefits for the early detection of lung cancer using TEPs, this technique has some limitations: (i) The need for more validation and standardization of assays. (ii) Difficulty in distinguishing TEPs from normal platelets. (iii) Detection techniques are not widely available. (iv) Problem of reproducibility with difficulties in capturing the full spectrum of tumor heterogeneity. (v) Time-consuming and expensive, which limits their clinical applicability [178,179,180].

**Table 2:** Summary of advantages and limitations of liquid biopsy biomarkers used in early lung cancer diagnosis.

Biomarkers	Advantages	Limitations	References
cfDNA and ctDNA	Genetic mutation and epigenetic alterations reflect those of the original tumor. Epigenetic changes are stable and occur in the early stage of cancer and represent tumor heterogeneity and dynamics. Availability of highly sensitive assays (PCR, NGS).	cfDNA is increased in some benign conditions such as cardiovascular diseases, other lung diseases, and infections. Utilization of this method is associated with high costs. The need for standardization of laboratory protocols and assays to achieve consistent and reproducible results. Individuals with early-stage malignancies have lower plasma levels of ctDNA.	[78,79,80,81,82,83,85,87,88,89,90]
miRNA	Different profiles among early-stage cancer patients. Stable in most types of body fluids (e.g., respiratory samples). Commercial kits available for collection.	High variability, according to patients and technologies, requiring normalization methods. Quantification and detection methods need to be validated. Unspecific for cancer subtype. RNAs’ molecular diagnostic accuracy for nonmalignant cases to the lung was not evaluated.	[9,106,107]
CTCs	Allows morphological and molecular characterization of lung cancer. High technical testing sensitivity available.	Sample sizes are very small, and data are limited in the majority of studies. CTCs are rare in blood in early-stage lung cancer. The observed heterogeneity of CTCs. Lack of standardized techniques that are validated and can be easily accessed and used.	[112,113,120,121]
TDEs	Carry specific biological information, as they comprise several types of biomarkers such as RNAs, DNAs, and proteins. Increased/decreased in lung cancer patients. Stable and accessible in most types of body fluids. Commercial kits available for isolation.	Efficient extraction and purification of exosomes is a challenge. Low repeatability and complex composition of exosomal RNAs. Require technical standardization and clinical validation to enhance the practical application. High costs.	[124,125,156,157]
TEPs	Carry tumor-modified RNA that reflects the tumor cells’ genetic alteration transcripts. Stable and highly abundant TEPs. TEPs are easily isolated in a clinical laboratory. Compatible with RNA-seq and AI pipelines.	The need for more validation and standardization of assays. Difficulty in distinguishing TEPs from normal platelets. Detection techniques not widely available. Problem of reproducibility with difficulties in capturing the full spectrum of tumor heterogeneity. Time-consuming and expensive, which limits their clinical applicability.	[175,176,177,178,179,180]

cfDNA = circulating cell-free DNA, CTCs = circulating tumor cells, ctDNA = circulating tumor DNA, miRNA = microRNA molecules, TDEs = tumor-derived exosomes, TEPs = tumor-educated platelets.

## 4. Disrupted Metabolic Pathways and Volatile Organic Compounds (VOCs)

Volatile organic compounds (VOCs) detected in exhaled breath air act as metabolic byproducts and represent biochemical fingerprints of the metabolic process in the body [181]. Many metabolic pathways are altered by malignant lung cancer cells. Rapid proliferation of cancer cells increases anaerobic oxidation and changes glycolysis metabolites with the accumulation of lactate and increases the consumption of phospholipids that form cell membranes [182]. Amino acids such as glutamine and serine are involved in the disrupted metabolic pathways. Glutamine is a significant nutrient source for malignant cells. Serine, as a precursor of purine, is involved in DNA synthesis and cancer cell proliferation [183]. Studies have shown that serum glutamine levels vary with the stage of lung cancer. It increases in the early stage of lung cancer secondary to stress response and decreases in advanced stages as patients frequently present with cachexia [184,185]. Also, serine is a key factor in cancer cell metabolism, and monitoring its metabolism is very important for studying the development of lung cancer [182]. These metabolic alterations in lung cancer metabolites are frequently described in other tumors and therefore lack specificity [186,187,188]. On the other hand, the altered metabolism of cancer cells results in the production of cancer-specific volatile organic compounds (VOCs) that have potential for early detection of the cancer [189,190]. Also, cancer cells often show higher rates of fatty acid metabolism. The breakdown of these fatty acids produces a variety of volatile byproducts, such as ketones (e.g., acetone) and aldehydes (e.g., hexanal). In addition, oxidative stress within the tumor microenvironment can cause the peroxidation of lipids in cell membranes. This process releases volatile hydrocarbons, such as pentane, isoprene, and 2-methylpentane [190].

### 4.1. VOC Detection Methods

VOCs are carbon-based molecules that are produced as byproducts of the cellular metabolic process and detected as a gas at room temperature [191]. Once formed, these VOCs diffuse into different body tissues and the circulatory system. They can consequently be detected in various body fluids and in exhaled air [191]. Recent studies showcased that VOCs’ analysis in blood, urine, saliva, feces, sweat, and exhaled air can be used effectively in detecting several types of cancer in the breast, prostate, colon, stomach, and skin (melanoma) [192,193,194,195,196,197]. “Respiratory biopsy” is the collection of VOCs from exhaled breath to detect diseases. Breath is collected through a non-invasive process into a device that can capture breath. The sample is then stored for analysis. VOCs in exhaled breath are being researched for early lung cancer detection, analyzing the unique VOC “fingerprint” produced by cancer cells [196,197].

Analytical techniques used to detect VOCs include gas chromatography with mass spectrometry (GC-MS), selected ion flow tube-mass spectrometry (SIFT-MS), proton-transfer-reaction mass spectrometry, infrared spectroscopy, or Quadrupole Time-of-Flight GC-MS (GC-MS QTOF) [198,199], with GC-MS being the most reliable technique [191]. Other methods for detecting VOCs include solid-state pattern recognition devices, i.e., electronic noses (e-noses). Although these devices cannot identify specific VOCs, they can detect malignancy patterns [200]. Recently, a miniature e-Nose system with 14 gas sensors from four different sensor array types has been described [201]. Use of this device, which is designed as a portable, cheap, user-friendly, and fast approach, has shown promise in detecting VOC patterns associated with lung cancer [200,202,203].

### 4.2. VOCs and Early Detection of Lung Cancer

VOCs measured in exhaled breath samples could differentiate between histologically confirmed lung cancer patients and healthy subjects with high sensitivity and specificity. One study primarily enrolled patients with locally advanced lung cancer. The selected ion flow-tube mass spectrometry (SIFT-MS) technique was used, showing high performance with a sensitivity of 96% and a specificity of 88% [204]. Another study that used gas sensor array response and included lung cancer patients with different stages also showed high performance with a sensitivity of 81% and a specificity of 91%; however, the sensitivity to lung cancer at stage I was higher with respect to stages II/III/IV (92% and 58%, respectively), indicating the potential of VOCs for early detection of the disease [205]. Several studies show that exhaled VOCs measured by various e-nose methods can successfully detect lung cancer patients and differentiate them from controls [206,207,208,209]. A meta-analysis of 25 studies that included 2045 lung cancer patients and 2200 controls showed that the sensitivity and specificity of lung cancer detection using VOCs in exhaled air are 85% and 86%, respectively, highlighting the potential of VOC measurement for early lung cancer detection [210].

### 4.3. Challenges Associated with Standardization of VOC Analysis in Lung Cancer

Standardization of VOC analysis in lung cancer is challenging due to variations in all phases of the process, including the following: (i) Sample collection and storage challenges due to contamination from the environment (sampling equipment, medical devices, or the storage containers used for breath samples), variable sampling methods (due to variations in breathing patterns and the time and duration of exhalation), and pre-analytical factors (patient activities, such as eating, drinking, or exercising, storage conditions, and handling of the sample prior to analysis). (ii) Laboratory analysis challenges due to inconsistent analytical techniques (GC-MS vs. SIF-MS), lack of standardized data processing (many studies use different algorithms and validation methods), and incomplete VOC fingerprinting (e-noses provide a general “fingerprint” of VOCs rather than identifying specific compounds). (iii) Biomarker validation challenges due to inconsistent biomarker lists (no universally validated list of VOC biomarkers for lung cancer), limited understanding of VOCs’ origin (while some VOCs may arise from cancerous cells, others could be a product of inflammation or other systemic changes), poor in vitro–in vivo correlation (in vitro studies using cancer cell lines often show poor correlation with VOCs found in patient breath samples), and small sample size studies (small sample size studies increase the risk of overfitting diagnostic models) [211].

Proposed solutions for these challenges and future directions include the following: (i) Establishing reference standards: It is very important to create a universal set of guidelines for breath collection and analysis, improving reproducibility and comparability across different research groups and clinical settings. (ii) Need for clinical trials: Large-scale clinical trials are needed to validate the diagnostic models of VOC analysis before integrating them into clinical practice. (iii) Comparison to other conditions: VOC profiles of lung cancer patients should be differentiated from those of healthy individuals and from other respiratory diseases, such as COPD, which may have similar VOC patterns. (iv) Improve data integration: To achieve more accurate and effective diagnostic models, VOC analysis could be combined with other traditional screening methods like LDCT scans and a patient’s full clinical profile [211,212,213].

### 4.4. VOCs’ Advantages and Limitations

The reported accuracies of exhaled VOC measurements in the early detection of NSCLC and/or pulmonary nodule management are favorably comparable with those of other biomarkers with the following advantages: (i) It is the least invasive technique, as samples are collected from exhaled breath, which is painless and does not require needles or biopsies. (ii) Convenient (easily collected), rapid, and cost-effective: The sample collection is fast, and the analysis can potentially be performed quickly at a lower cost than imaging techniques. These factors increase patient compliance and encourage greater patient participation in screening initiatives. (iii) The only technique that gives near real-time results. (iv) Compatible with AI pipelines. (v) Point-of-care potential (eNose) [214,215]. However, this technique has challenges associated with standardization that lead to poor reproducibility and difficulty in comparing results between studies (see Section 4.3).

## 5. Artificial Intelligence (AI)

Artificial intelligence (AI) involves the utilization of machine learning models for the analysis of medical data obtained from different sources to achieve additional insights to improve patient care management. Biomedical data are increasing in volume and complexity, which makes AI valuable for extracting clinically meaningful patterns, particularly in situations where signals are subtle or noisy. Using AI-driven algorithms together with diagnostic modalities can enhance the interpretation of the complex data of these modalities and minimize the interobserver variability associated with them [216]. AI-driven analysis becomes very important for early detection of lung cancer when traditional diagnostic modalities like imaging, molecular assays, and biopsies become inadequate because of invasiveness, small amounts at early stages, or high costs [217]. AI-powered computer-aided detection systems help in detecting pulmonary nodules with increased sensitivity and reducing the false positives associated with some diagnostic methods, such as LDCT, leading to earlier and more accurate diagnoses [218]. Combining AI with the different lung cancer screening modalities can further enhance early detection of the disease.

### 5.1. AI-Based LDCT

Over the past five years, thousands of lung cancer patients and far more controls were included in many studies exhibiting excellent effectiveness of AI-based chest CT as a second reader in detecting lung cancer or differentiating between benign and malignant lung nodules with an accuracy of about or more than 90% and nearly 100% in some cases [219]. The combination resulted in a substantial reduction in the number of false-negative and false-positive cases [219], demonstrating better results than that of radiologists. However, there were cases in which AI-based chest CT was not as good as the radiologists’ [220] and in other cases failed to achieve accuracy like that achieved by skilled radiologists, suggesting that the role of AI in screening for lung cancer should be supplementary rather than a leading one [221]. AI-based chest CTs can provide a significant reduction in radiation dosage, which is very important for screening programs. Their accuracy for solid or sub-solid nodules was not affected by extreme dosage reduction of 15–76% [222,223], and their accuracy for ground glass nodules was not affected by 10- to 20-fold dose reduction [224]. The diagnostic accuracy for pulmonary lesions is not affected by the reduction in radiation between 0.07 and 0.14 mSv. AI-based algorithms with ultra-low doses of radiation were successful in detecting lung cancer in several studies [224,225]. The implementation of AI-based LDCT screening has been shown to be cost-effective, as it reduces false-negative cases and costs of their added future treatments, as well as false-positive cases and costs of their needless follow-ups [226]. In addition, AI-based screening programs for detecting lung cancer can be enhanced by epidemiological characteristics, including age, gender, family history of cancer, history of emphysema, and the use of biomarkers [219,227,228,229,230], such as liquid biopsy biomarkers and other serum tumor markers [228,231,232,233]. The LDCT scans of these AI-based lung screening programs can reliably detect and provide information about other conditions like coronary artery calcification, aortic aneurysms, osteoporosis, masses in the neck, thorax, or abdomen, emphysema, interstitial lung disease, and pulmonary fibrosis [234,235].

### 5.2. AI-Based Liquid Biopsy Biomarkers

AI has become invaluable in overcoming the liquid biopsy limitation of the presence of a low quantity of its biomarkers in early-stage lung cancer. AI and liquid biopsy work together to enable non-invasive, earlier detection of lung cancer by analyzing molecular biomarkers [236]. Liquid biopsy detects these markers, while AI algorithms analyze the complex data to identify subtle patterns and mutations indicative of early-stage cancer, offering higher accuracy. It was shown that the application of a deep-learning model to cfDNA methylation with fragment-size profiles could differentiate between lung cancer patients and healthy subjects with an 81.5% accuracy [236]. Also, it was demonstrated that applying deep learning models to circulating tumor cell-derived RNA (ctcRNA)-based tissue deconvolution could trace the original tissue of metastatic tumors, thus highlighting the potential clinical significance of this approach in early detection of cancer metastasis [237]. This integrated approach of combining AI and liquid biopsy has the potential to transform lung cancer screening from a passive, symptom-driven process to an active, personalized, and preventive one, improving patient outcomes and survival rates.

### 5.3. AI-VOCs

The conventional univariate/rule-based VOC methods rely on predefined rules and thresholds for single compounds, offering simplicity but struggling with complexity and adaptability. Conversely, AI-based VOC methods use machine learning (AI) to analyze complex patterns across the VOCs’ biochemical fingerprints simultaneously, capturing subtle changes that may not be apparent through univariate/rule-based methods and providing higher accuracy and efficiency in applications like disease detection [238]. This was presented in a recent study where gas chromatography field asymmetric ion mobility spectrometry (GC-FAIMS) was used to analyze exhaled breath air samples from lung cancer patients and healthy subjects. In this study, the neural network architectures GoogLeNet and VGG-11 were trained on exhaled breath air sample spectrograms and could differentiate lung cancer patients from healthy subjects with an average accuracy of 84% and 81%, respectively [238]. Similarly, another more recent study showed that AI-based VOC profiles utilizing gas chromatography coupled to ion-mobility spectrometry (GC-IMS) allowed lung cancer detection with an accuracy of 90%, a sensitivity of 87%, and a specificity of 92% without the need for qualitative information about specific biochemical components [239]. These results support the potential of AI-based VOC methods in revolutionizing early lung cancer detection.

### 5.4. AI-Based Multimodality Strategies

**Figure 3:**
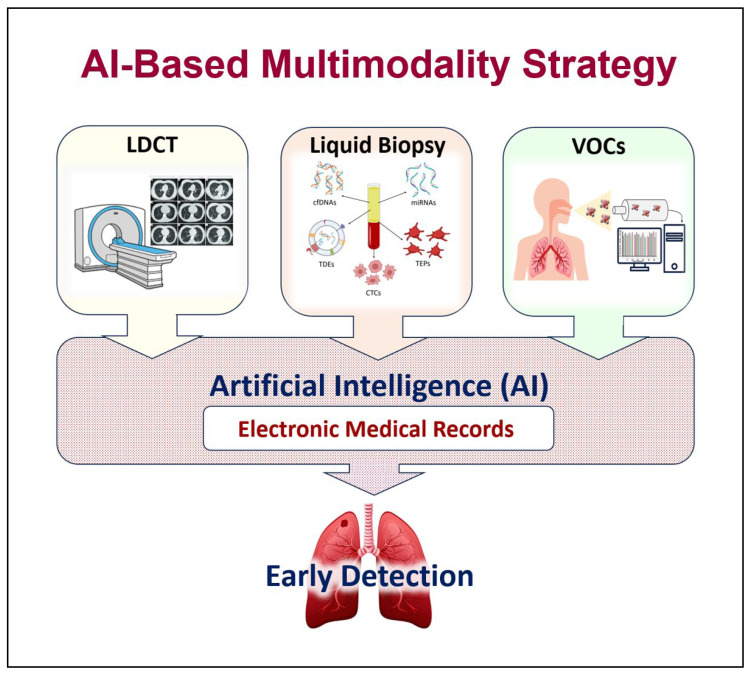
Artificial intelligence (AI)-based multimodality strategy. Combining AI with different lung cancer screening modalities can further enhance early detection of the disease. (LDCT = low-dose computed tomography, EB-OCT = endobronchial optical coherence tomography, VOCs = volatile organic compounds).

With the current advanced technologies, substantial amounts of data related to LDCT imaging techniques and to liquid biopsy biomarkers are generated. Analysis strategies based on AI play a key role in extracting and analyzing the quantitative features of the LDCT images and in obtaining large amounts of data from the different liquid biopsy biomarkers. AI can extract large quantitative features (radiomics) from the LDCT images, which are qualitative interpretations. Similarly, it can permit the study of the protein and nucleic acid biomarkers of liquid biopsies (proteomics/genomics) [217] (Figure 3).

AI uses various algorithmic methodologies for early lung cancer detection, primarily based on machine and deep learning, to analyze medical data, especially imaging scans. AI models for early lung cancer detection are trained to identify and characterize pulmonary nodules from imaging and other patient data. Imaging-based methods include the following: (i) Convolutional neural networks (CNNs): This is the most common deep learning approach for analyzing medical images like LDCT scans and X-rays. CNNs are highly effective at detecting pulmonary nodules and classifying them as benign or malignant. (ii) Computer-aided detection (CAD) systems: Initially, CAD systems used traditional machine learning to mark suspicious areas for a radiologist’s review. Current versions are usually powered by deep learning. They act as “second readers” to increase detection rates and reduce the radiologist’s workload. (iii) Radiomics technique: It extracts and analyzes high-dimensional features from medical images that are not visible to the human eye. Machine learning models, such as support vector machines or logistic regression, can then use these features to predict the malignancy of a nodule. (iv) Integrated imaging: This technique combines AI analysis of LDCT scans with other imaging like PET-CT scans and can further improve diagnostic accuracy by incorporating metabolic activity into the model [217,240]. On the other hand, non-imaging-based methods include the following: (i) Biomarker models: These can analyze panels of blood-circulating biomarkers (liquid biopsy) or exhaled VOCs (respiratory biopsy) to non-invasively identify cancer. (ii) Clinical data models: These are machine learning algorithms that can predict lung cancer risk by analyzing a patient’s demographic information, clinical history, and environmental risk factors. Finally, integrated methods include the following: (i) AI-based multimodalities: These hybrid systems that combine data from imaging, genomics, and clinical records can provide more comprehensive assessments. (ii) Multi-omics integration modalities: These combine radiomics data with molecular-level information, including genomics (gene mutations like EGFR), transcriptomics (RNA expression), proteomics (protein expression), and epigenetics [217,240].

Recently, the ASCEND-LUNG trial, a prospective multimodality study, integrated a multi-omics liquid biopsy-based model formed on cfDNA methylation and protein features for early lung cancer screening and AI-aided LDCT analysis to discriminate between benign and malignant pulmonary nodules. The study highlighted the success and validation of the integrated system that was developed, which might provide a potentially applicable solution for early lung cancer detection [241]. AI-based multimodality models have gained more attention recently as they could revolutionize early lung cancer screening, especially if combined with clinical information (smoking history and other risk factors) [242,243].

### 5.5. AI’s Ethical Considerations

The deployment of AI in early lung cancer detection raises critical ethical concerns to ensure fair, safe, and transparent implementation: (i) Data privacy and security: AI models require access to large amounts of sensitive patient data for training. Protecting this information from breaches is a significant concern. This requires strong legal frameworks and technical safeguards, such as federated learning and encryption. (ii) Algorithmic bias: AI models may not perform optimally in diverse populations if the training data is not representative. For example, training on a dataset with a limited variety of tumor sizes may lead to poor detection of smaller tumors. (iii) Transparency and explainability: Many deep learning models operate as “black boxes” where the reasoning behind their output is not easily understood. This lack of transparency can hinder trust among clinicians and patients. Clinicians need interpretable AI to understand and validate the system’s recommendations, ensuring patient safety and informed decision-making. (iv) Accountability and liability: Vagueness exists regarding who is responsible for a medical error caused by an AI system. The lack of clear regulations makes it difficult to assign blame to the manufacturer, hospital, or clinician, potentially undermining patient safety. (v) Patient autonomy and informed consent: The process of obtaining informed consent becomes more complex when AI is involved. Patients need to understand the potential benefits and harms of AI-assisted diagnosis. The risk of overdiagnosis and unnecessary procedures is a critical factor for shared decision-making. (vi) Stigma and inequity: Lung cancer is often associated with the stigma of smoking. AI-based risk models that rely on smoking history may perpetuate or amplify this stigma. Programs must also ensure equitable access, as those from lower socioeconomic groups may be less likely to participate despite being at a higher risk [240,244,245].

### 5.6. Implementation Challenges

The transition of AI-powered early detection tools from research to clinical practice faces several organizational and logistical hurdles. (i) Clinical integration and workflow: Integrating AI systems into existing hospital workflows, including electronic health records and radiology reporting, can be difficult due to platform incompatibility and a lack of interoperability. Providers may also resist technologies that disrupt established clinical routines or require specialized training. (ii) Validation and standardization: Many AI studies are retrospective and lack the real-world validation needed for clinical use. A lack of standardized datasets and assessment methods makes it difficult to compare the performance of different AI models. (iii) Resource and infrastructure demands: Implementing AI requires substantial investment in computational hardware and data infrastructure. Many healthcare systems lack the resources, including staffing and equipment, to support this technology at scale, particularly in underserved regions. (iv) Clinician education and training: This depends on buy-in and proficiency from healthcare professionals. Medical and technical staff require training to use, interpret, and validate AI outputs effectively for successful integration. This demands an overhaul of existing medical education and continuing professional development. (v) Cost-effectiveness: While AI promises long-term cost savings through improved efficiency, the initial costs of development, validation, and implementation can be prohibitive. Cost-effectiveness is dependent on many factors, including the target population, screening intervals, and downstream treatment costs. (vi) Managing false positives: Although AI can increase sensitivity, it can also raise the rate of false positives, leading to patient anxiety, unnecessary follow-up procedures, and increased healthcare costs. Nodule management protocols must be carefully optimized to minimize these harms [244,245,246].

### 5.7. Proposed Solutions and Strategies

Proposed solutions and strategies to overcome implementation challenges for AI in early lung cancer diagnosis include the following: (i) Engaging with domain experts and diverse stakeholders by involving clinicians, data scientists, patients, and other stakeholders from the initial stages of development to ensure the AI tool meets clinical needs, avoids bias, and maintains ongoing dialogue to foster trust and adapt the technology to real-world use cases. (ii) Reviewing and applying ethical frameworks by establishing and adhering to ethical principles. The aim is to guide development, addressing data privacy and security through technical solutions and clear policies. This is achieved by conducting thorough pre- and post-deployment audits to monitor for bias, fairness, and clinical validity across all subgroups. (iii) Proposing and conducting pilot studies by implementing them in a clinical setting to test AI performance in a real-world environment before widespread adoption. These studies can be used to gather data on clinical workflow integration, user trust, and cost-effectiveness, ensuring external validation and adherence to reporting guidelines. (iv) Developing and refining AI technology by emphasizing multimodal AI approaches that combine imaging, clinical, and molecular data. These combinations have shown superior performance, prioritizing the use of explainable AI (XAI) models to increase transparency, build clinician confidence, and develop models on large, multicenter, and demographically diverse datasets to improve generalizability and fairness [240,247,248].

### 5.8. Future Perspectives

Future perspectives for AI-based multimodality strategies in early lung cancer diagnosis include integrating AI with new screening methods like liquid biopsies and VOCs, creating personalized risk-stratified screening, and developing more sophisticated models that combine imaging data, genetic profiles, and clinical history. These strategies promise to improve the accuracy and speed of diagnosis, distinguish between benign and malignant nodules, and guide more effective personalized treatment plans [244].

In the area of enhanced screening and detection, AI will enable further reduction in radiation LDCT scans by improving image reconstruction while maintaining image quality [242]. Integration of AI with liquid biopsies/VOCs could significantly improve the sensitivity and specificity of detecting early-stage cancers that traditional methods miss [242]. AI-powered CAD systems will function as a second reader for radiologists, improving accuracy and speeding up the interpretation of scans. AI will also enable the creation of personalized screening programs by analyzing a large amount of imaging, clinical data, and genetic profiles to better assess an individual’s risk. This risk stratification will enable more targeted and effective screening interventions for high-risk individuals [249].

Also, AI will move beyond simple detection to automatically segment and characterize suspicious nodules by assessing size, volume, and densitometric features. Advanced “virtual biopsy” models will be created by extracting radiomic features from imaging data to characterize abnormalities, potentially replacing or supplementing traditional biopsies [249]. AI will improve the ability to classify different lung cancer subtypes from imaging data with high accuracy [244].

In addition, AI will integrate multiple imaging modalities with clinical data, genomic profiles, and other biomarkers for a more comprehensive diagnostic assessment. This holistic approach will provide a more complete picture of the disease, leading to more accurate diagnoses and predictions [250,251].

Finally, AI will be used to predict treatment responses based on genetic profiles and other data, helping to tailor therapies for individual patients. It will aid in optimizing radiation therapy and predicting prognosis, including risk of recurrence, to inform better treatment decisions [249].

## 6. Conclusions

Despite their advantages, early lung cancer detection techniques have limitations that restrict their widespread use as individual public screening tools. Integration of AI with each of these techniques could provide significant advancements and offer potential solutions to challenges related to early detection of lung cancer. AI-based multimodality strategy involves extracting large quantitative features from the LDCT images (radiomics), which will help in improving nodule detection, risk stratification, predicting future cancer risk, and automating/streamlining radiology workflows (real-time decision support). Liquid biopsy and/or VOC biomarkers combined with clinical and radiologic data will help improve sensitivity and specificity. Therefore, adopting AI-based multimodality strategies, such as multi-omics liquid biopsy and/or VOCs’ detection, with LDCT augmented by advanced AI, could revolutionize early lung cancer screening by improving accuracy, efficiency, and personalization, especially when combined with patient clinical data. However, the successful integration of these technologies into clinical practice requires cross-disciplinary collaboration to validate and implement these innovative AI-based multimodal strategies by integrating expertise from oncology, data science, radiology, and pathology. This collaboration is needed to overcome challenges in data integration, overcome implementation barriers like user interface and workflow integration, and ensure clinical validation through large-scale studies.

## Figures and Tables

**Table 1:** Current (2025) screening recommendations of the National Comprehensive Cancer Network (NCCN) and the European Society of Thoracic Imaging (ESTI) for management of solid nodules [22,47].

Recommendation	NCCN—2025	ESTI—2025
Baseline screening		
12-month CT	<6 mm	<100 mm^3^ (<6 mm) +No suspicious features
6-month CT	≥6 to <8 mm	≥100 to <250 mm^3^ (≥6 to <8 mm) +No suspicious features <100 mm^3^ (<6 mm) +Suspicious features
3-month CT	≥8 to <15 mm ≥15 mm +Low suspicion of lung cancer	≥250 to <500 mm^3^ (≥8 to <10 mm) +No suspicious features ≥100 to <250 mm^3^ (≥6 to <8 mm) +Suspicious features
1-month CT	NA	≥500 mm^3^ (≥10 mm) +No suspicious features
Referral for Workup	≥15 mm +High suspicion of lung cancer	≥250 mm^3^ (≥8 mm) +Suspicious features
Repeat rounds screening		
12-month CT	Prevalent nodule Unchanged and <8 mm Unchanged and ≥8 mm to <15 mm and unchanged after 6 months New nodule <4 mm	Prevalent nodule VDT ≥ 400 d after 6 months OR VDT ≥ 500 d after 12 months Average diameter increase ≤1.5 mm/year New nodule <30 mm^3^ (<4 mm)
6-month CT	Prevalent nodule Unchanged and ≥8 mm to <15 mm Unchanged and ≥15 mm +Low suspicion of lung cancer New nodule 4 mm to <6 mm	Prevalent nodule VDT ≥ 250 d after 3 months Average diameter increase ≤ 1.5 mm/3 months
3-month CT	Prevalent nodule Growing (>1.5 mm) and <8 mm Growing (>1.5 mm) and ≥8 mm +Low suspicion of lung cancer New nodule 6 mm to <8 mm ≥8 mm +Low suspicion of lung cancer	New nodule ≥30 mm^3^ (≥4 mm)
Referral for Workup	Prevalent nodule Unchanged and ≥15 mm +High suspicion of lung cancer Growing (>1.5 mm) and ≥8 mm +High suspicion of lung cancer New nodule ≥8 mm +High suspicion of lung cancer	Prevalent nodule Total diameter growth >5 mm VDT < 250 days after 3 months OR VDT < 400 days after 6 months OR VDT < 500 days after 12 months Average diameter increase >1.5 mm/year New nodule follow-up >15% volume growth at 3 months >1.5 mm diameter growth at 3 months

NA = not available, VDT = volume doubling time.

## Data Availability

Not applicable. No new data were created or analyzed in this study.

## Abbreviations

The following abbreviations are used in this manuscript:

ACS	American Cancer Society
AI	Artificial intelligence
CAD	Computer-aided detection
cfDNA	Circulating cell-free DNA
CNNs	Convolutional neural networks
circRNA	Circular RNAs
ctcRNA	Circulating tumor cell-derived RNA
CTCs	Circulating tumor cells
ctDNA	Circulating tumor DNA
CXR	Chest X-ray
DANTE	Detection And screening of early lung cancer with Novel imaging Technology
DLCST	Danish Lung Cancer Screening Trial
EGFR	Epidermal growth factor receptor ()
ESTI	European Society of Thoracic Imaging
GC-MS	Gas chromatography with mass spectrometry
ITALUNG	Italian Lung study
LCS	Lung cancer screening
LDCT	Low-dose computed tomography
lncRNA	Long non-coding RNA
Lung-RADS	Lung CT Screening Reporting and Data System
LUSI	German Lung Cancer Screening Intervention
MILD	Multicentric Italian Lung Detection trial
miRNA	MicroRNA molecules
NCCN	National Comprehensive Cancer Network
NELSON	Nederlands-Leuvens Longkanker Screenings Onderzoek trial
NLST	National Lung Screening Trial
NSCLC	Non-small-cell lung cancer
SIFT-MS	Selected ion flow tube-mass spectrometry
TDEs	Tumor-derived exosomes
TEPs	Tumor-educated platelets
UKLS	UK Lung Cancer Screening
ULDCT	Ultra-low-dose CT
USPSTF	United States Preventive Services Task Force
VDT	Volume doubling time
VOCs	Volatile organic compounds
